# Supplementary material for: Nanopore event detection in a simple and adaptive way
Source: bioRxiv. 2026 May 11:2026.05.07.723187. Preprint. [Version 1] doi: 10.64898/2026.05.07.723187 (PMC13192963; doi:10.64898/2026.05.07.723187)
Supplement: Supplement 1 [file media-1.pdf]

Supplementary Information - Nanopore event detection in a simple and adaptive way

Peijia Wei and Mayukh Kansari
Computational Biotechnology, RWTH Aachen University, Worringerweg 3, 52074 Aachen, Germany

Michael Mierzejewski
NMI Natural and Medical Sciences Institute at the University of Tübingen, 72770 Reutlingen, Germany

Tobias Ensslen
Laboratory for Membrane Physiology and Technology, Department of Physiology II, Faculty of Medicine, University of Freiburg, Hermann-Herder-Str. 7, 79104 Freiburg, Germany and Present address: Hahn-Schickard Institute for Microanalysis Systems, Georges-Koehler Allee 304, 79110 Freiburg i.Br.

Chih-Yuan Lin and Kyril Kavetsky
Department of Physics and Astronomy, University of Pennsylvania, Philadelphia, Pennsylvania 19104, United States

Peter Jones
NMI Natural and Medical Sciences Institute at the University of Tübingen, 72770 Reutlingen, Germany

Jan C. Behrends
Laboratory for Membrane Physiology and Technology, Department of Physiology II, Faculty of Medicine, University of Freiburg, Hermann-Herder-Str. 7, 79104 Freiburg, Germany

Marija Drndić
Department of Physics and Astronomy, University of Pennsylvania, Philadelphia, Pennsylvania 19104, United States

Maria Fyta
Computational Biotechnology, RWTH Aachen University, Worringerweg 3, 52074 Aachen, Germany and Center for Computational Life Sciences, RWTH Aachen University, Pauwelstrasse 19, 52074 Aachen, Germany

(Dated: May 7, 2026)

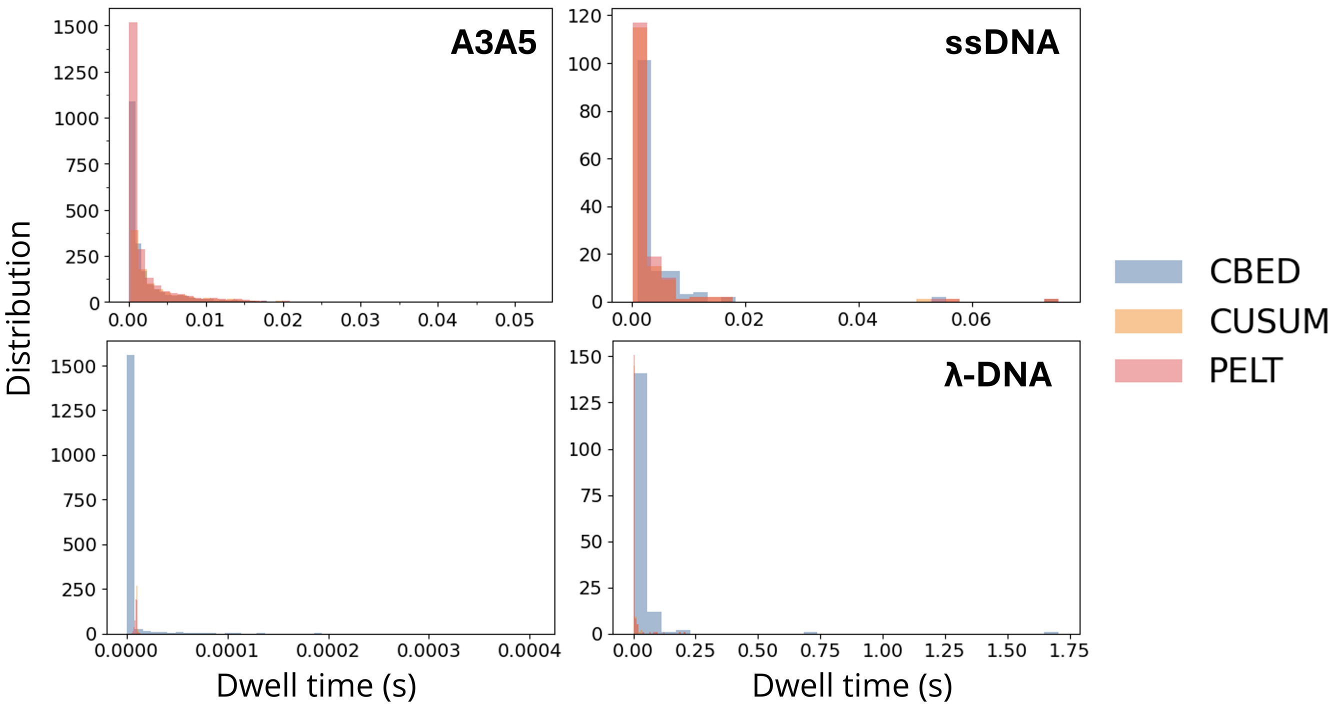

FIG. 1. Distributions of extracted event features dwell time for the A3A5, the ssDNA, the POC and the λ-DNA dataset, detected by CBED (blue), CUSUM (orange), and PELT (red).

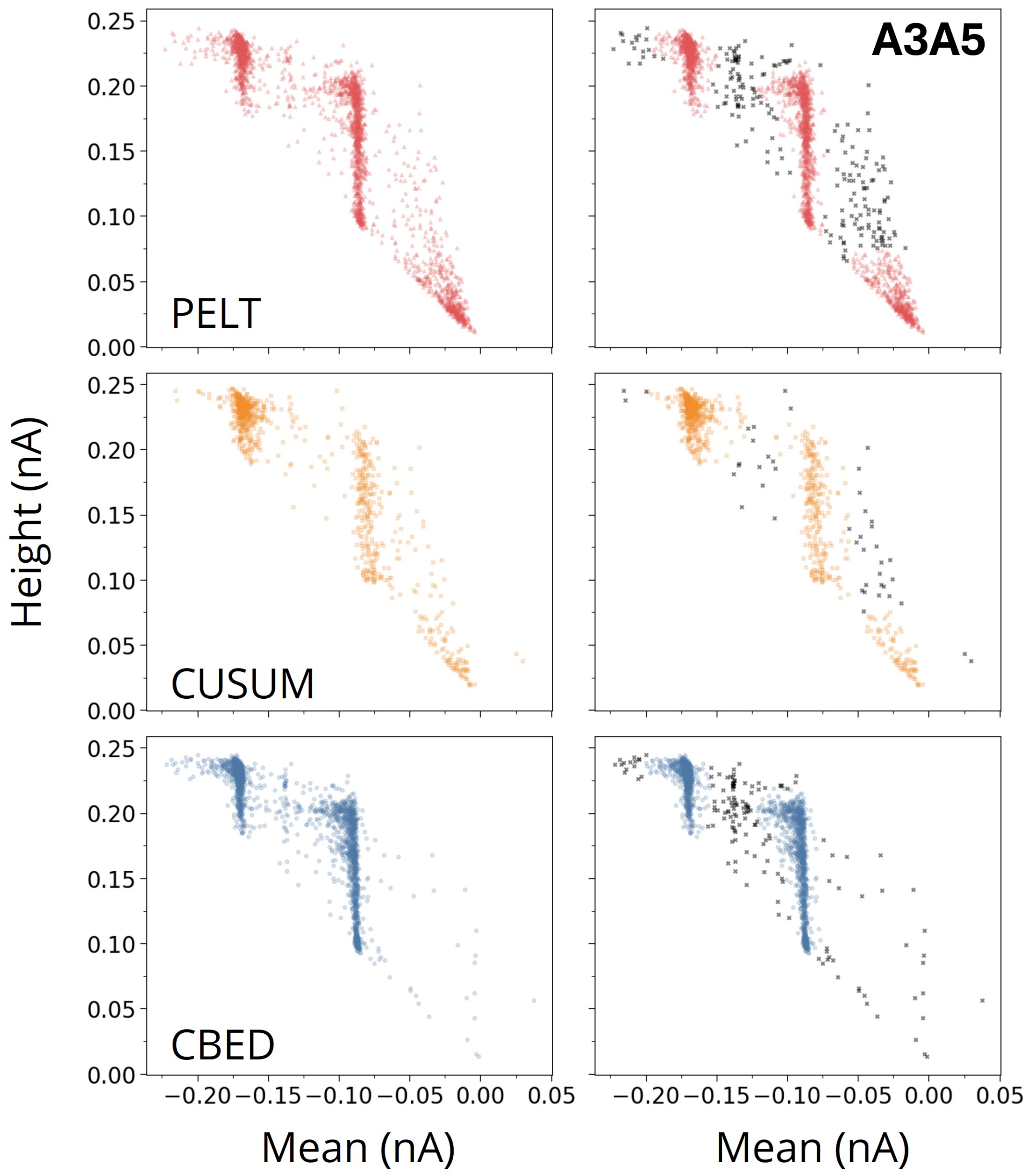

FIG. 2. Mean–depth clustering of A3A5 events. Results from K-means (left) and DBSCAN (right) for events detected by PELT, CUSUM, and CBED.

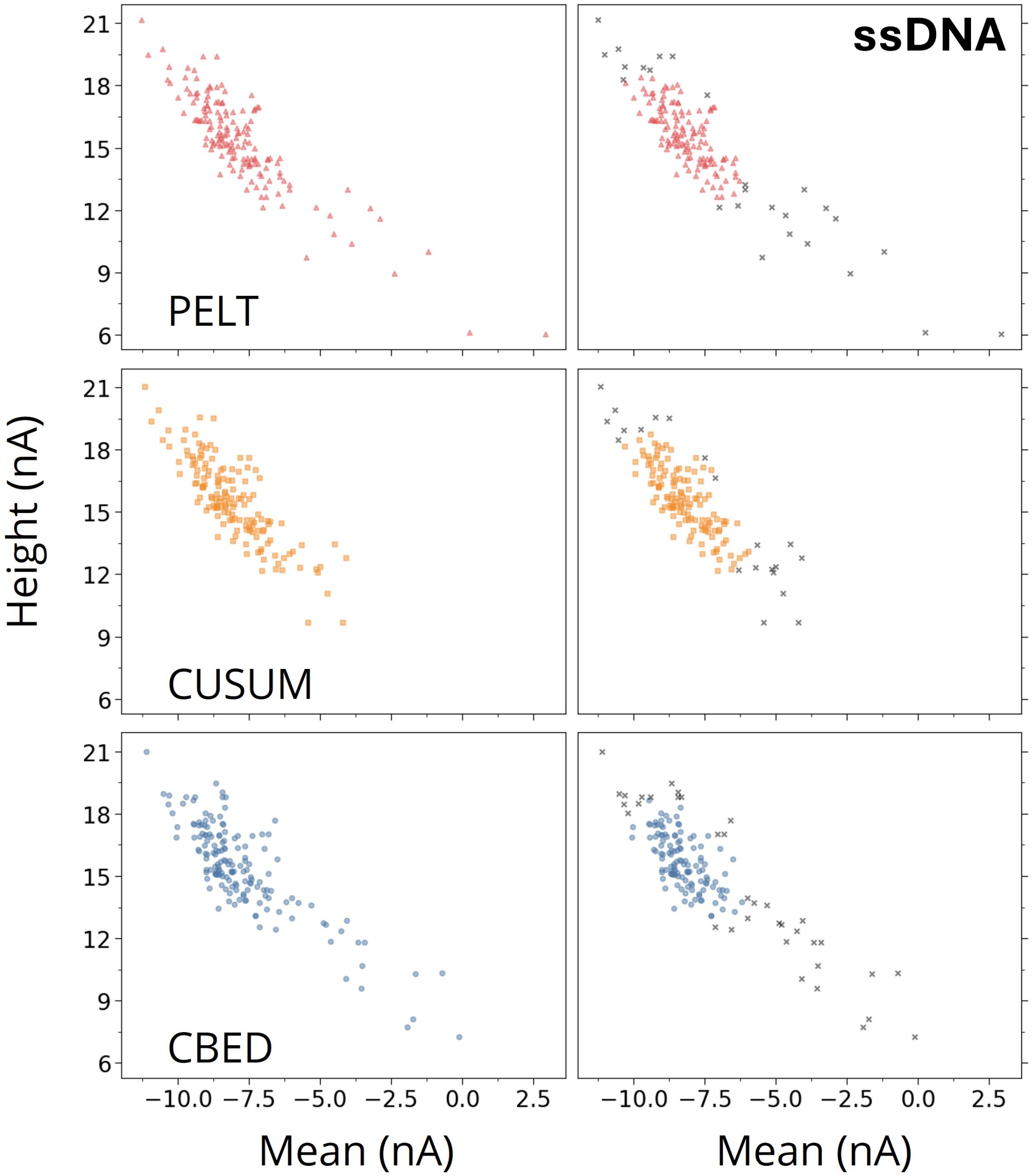

FIG. 3. Mean-depth clustering of ssDNA events. Results from K-means (left) and DBSCAN (right) for events detected by PELT, CUSUM, and CBED.

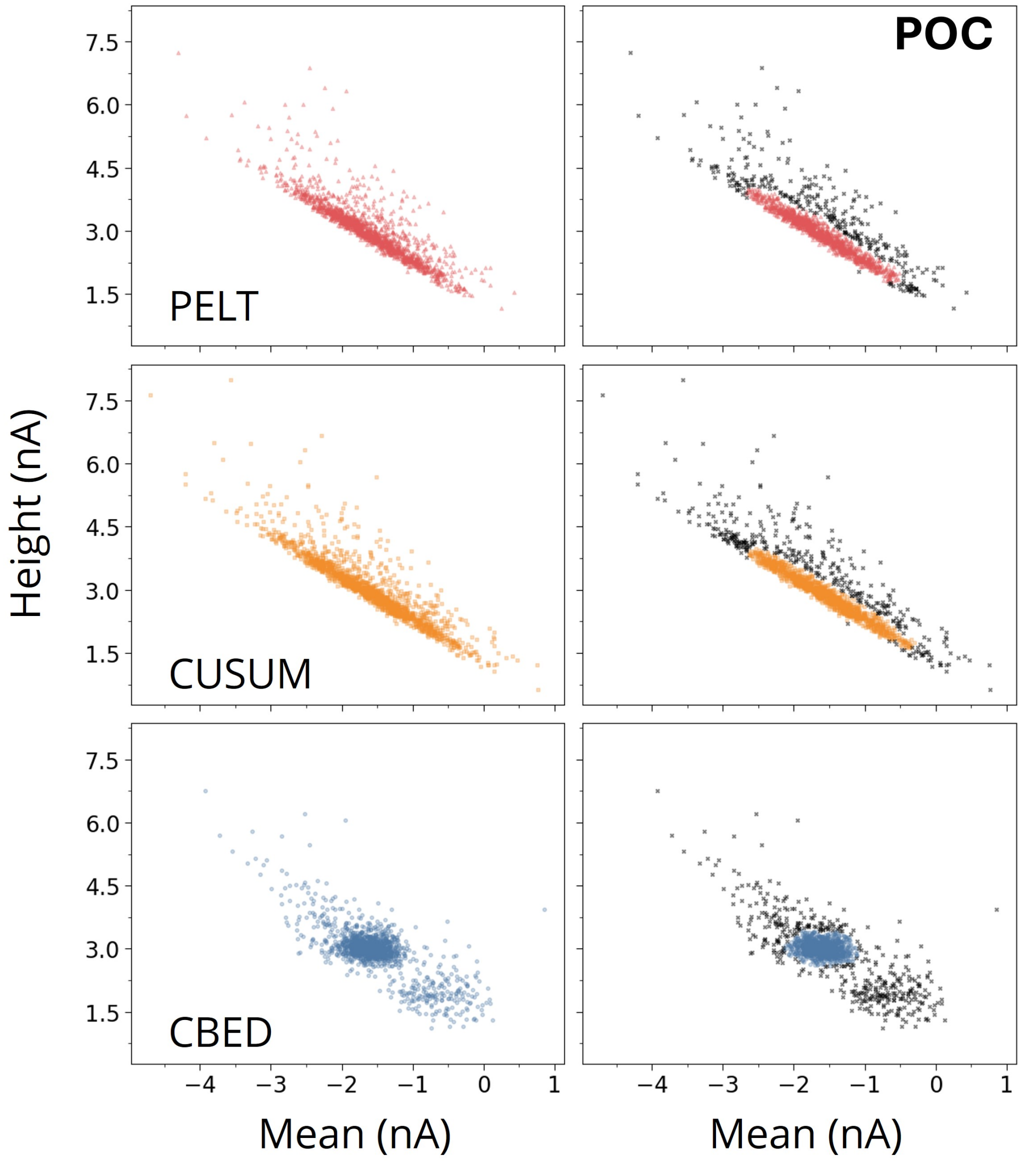

FIG. 4. Mean-depth clustering of POC events. Results from K-means (left) and DBSCAN (right) for events detected by PELT, CUSUM, and CBED.

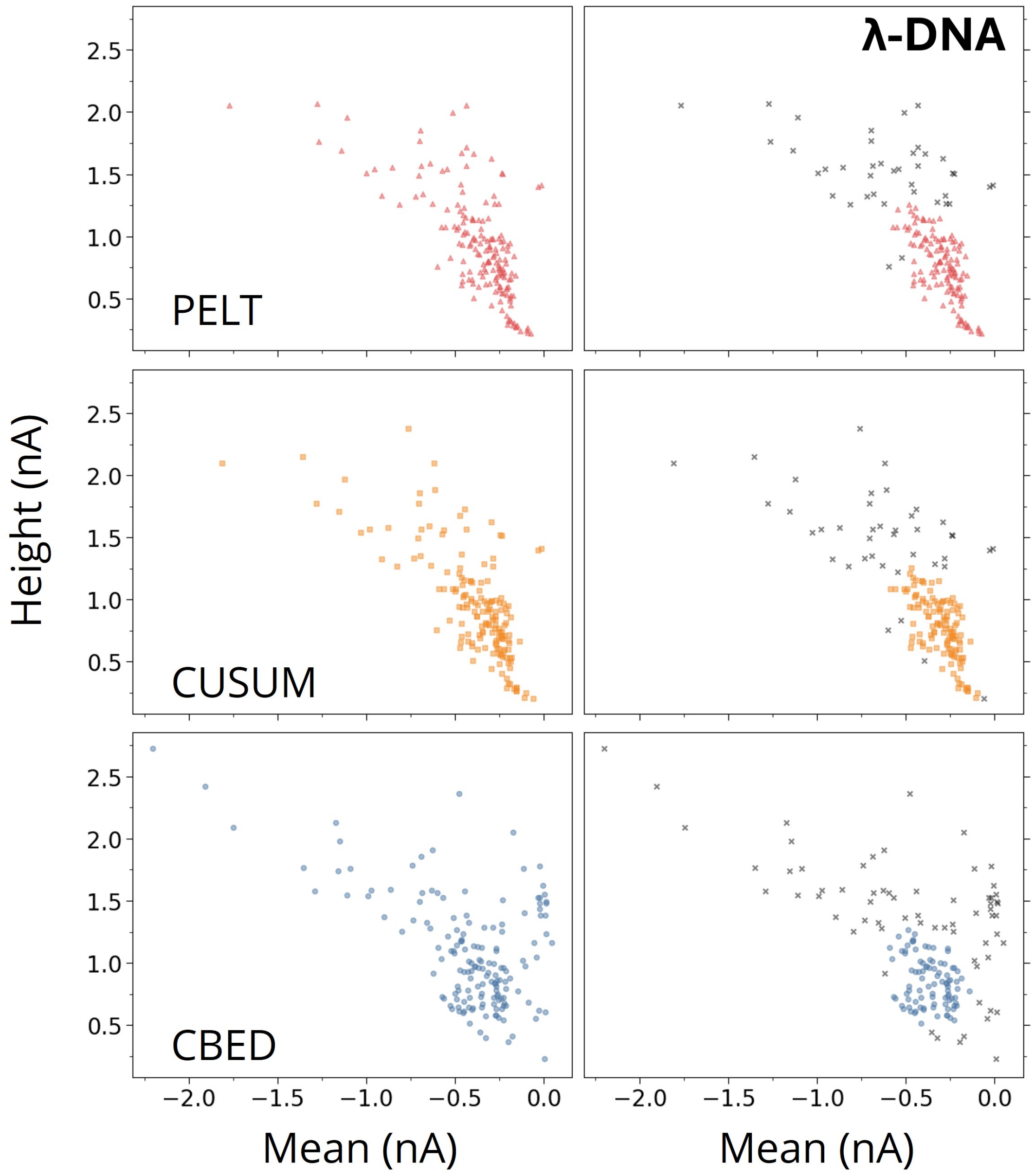

FIG. 5. Mean-depth clustering of λ-DNA events. Results from K-means (left) and DBSCAN (right) for events detected by PELT, CUSUM, and CBED.

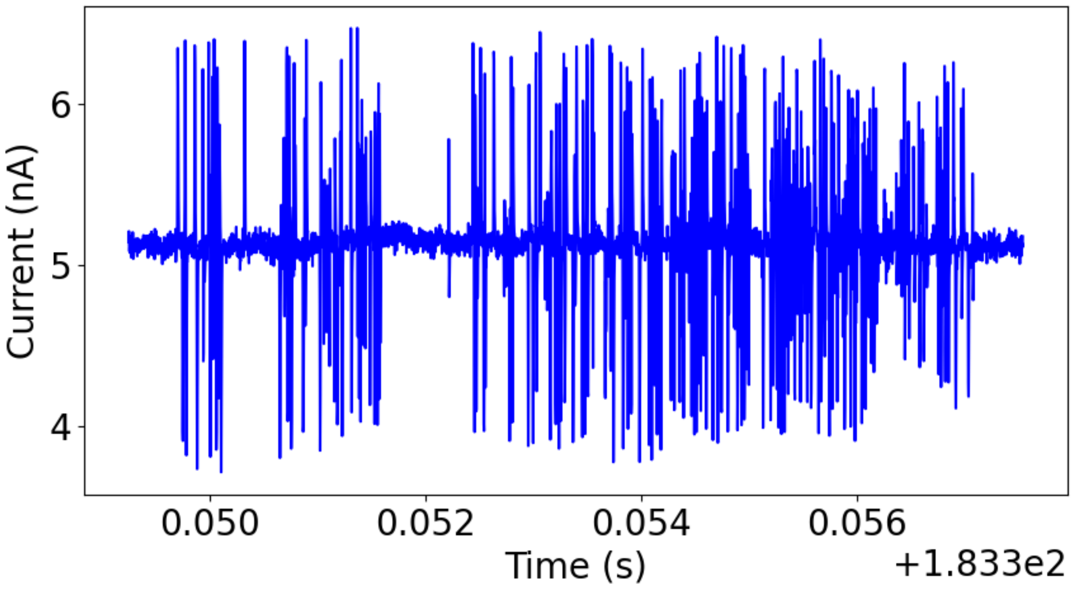

FIG. 6. Noise_λ-DNA_CBED.
